# The intensity of physiological and behavioral responses of horses to predator vocalizations

**DOI:** 10.1186/s12917-020-02643-6

**Published:** 2020-11-10

**Authors:** Iwona Janczarek, Anna Stachurska, Witold Kędzierski, Anna Wiśniewska, Magdalena Ryżak, Agata Kozioł

**Affiliations:** 1 Department of Horse Breeding and Use, University of Life Sciences in Lublin, Akademicka 13 str, 20-950 Lublin, Poland; 2 Department of Biochemistry, University of Life Sciences in Lublin, Lublin, Poland; 3 Institute of Agrophysics, Polish Academy of Sciences, Lublin, Poland

**Keywords:** horse, predator, vocalization, physiological response, behavior

## Abstract

**Background:**

Predatory attacks on horses can become a problem in some parts of the world, particularly when considering the recovering gray wolf populations. The issue studied was whether horses transformed by humans and placed in stable-pasture environments had retained their natural abilities to respond to predation risk. The objective of the study was to determine the changes in cardiac activity, cortisol concentrations, and behavior of horses in response to the vocalizations of two predators: the gray wolf (*Canis lupus*), which the horses of the breed studied had coevolved with but not been exposed to recently, and Arabian leopard (*Panthera pardus nimr*), from which the horses had been mostly isolated. In addition, we hypothesized that a higher proportion of Thoroughbred (TB) horse ancestry in the pedigree would result in higher emotional excitability in response to predator vocalizations. Nineteen horses were divided into groups of 75%, 50% and 25% TB ancestry. The auditory test conducted in a paddock comprised a 10-min prestimulus period, a 5-min stimulus period when one of the predators was heard, and a 10-min poststimulus period without any experimental stimuli.

**Results:**

The increase in heart rate and saliva cortisol concentration in response to predator vocalizations indicated some level of stress in the horses. The lowered beat-to-beat intervals revealed a decrease in parasympathetic nervous system activity. The behavioral responses were less distinct than the physiological changes. The responses were more pronounced with leopard vocalizations than wolf vocalizations.

**Conclusions:**

The horses responded with weak signs of anxiety when exposed to predator vocalizations. A tendency towards a stronger internal reaction to predators in horses with a higher proportion of TB genes suggested that the response intensity was partly innate. The more pronounced response to leopard than wolf may indicate that horses are more frightened of a threatening sound from an unknown predator than one known by their ancestors. The differing response can be also due to differences in the characteristic of the predators’ vocalizations. Our findings suggested that the present-day horses’ abilities to coexist with predators are weak. Hence, humans should protect horses against predation, especially when introducing them into seminatural locations.

## Background

Predatory attacks on horses can become a problem in some parts of the world, particularly when considering the recovering gray wolf (*Canis lupus*) population [1, 2]. Across millions of years of evolution, the survival of equids has depended on their capacity to adapt to their environment. Predators that threaten or kill ungulates could be an important factor limiting the development and migration of equid populations [3]. In open grasslands, equids could typically detect the presence of predators by sight, smell, and/or hearing. The antipredator defense response in horses is to flee from a fear-inducing cue. A weaker response may be to discontinue feeding and to become increasingly vigilant [4]. Frightening stimuli also induce physiological changes, such as increases in heart rate (HR) and cortisol concentrations [5–7].

Over the millennia since domestication, the consequent selection and emergence of different breeds in recent centuries have dramatically changed horses compared to their wild equid ancestors. Present-day horses can generalize disturbance stimuli that may seem to them to be analogous to predation risk; for instance, they can categorize humans as predators [8–11]. On the other hand, the antipredator response may well have been attenuated in present-day horses [12]. Modern horses have been selected for specific utility traits enabling safe human-horse cooperation [13]. Acute responses to fear-inducing cues had to be reduced. From a scientific point of view, it is interesting to assess whether the horses transformed by humans and placed in stable-pasture environments that are usually free of predators have retained their natural abilities to respond to predation risk in their habitats. The analysis may help to understand horse adaptations and indicate implications for better accommodating those adaptations to the changing environment.

The Polish Halfbred horse breed consists of crosses of Polish and foreign warmblood horses of various breeds. Indigenous horses mated to different imported horses, including Thoroughbred (TB) horses, were the origin of Polish warmblood breeds [14, 15]. TB horses are a strongly transformed breed compared to their wild ancestors. Many studies have indicated that TBs exhibit a high level of sensitivity and nervousness, which may be connected with the long single-trait selection for speed and the fact that the population has been closed for a number of generations [16–19]. Warmblood horses with a high proportion of TB ancestry also show more intense responses to various stimuli than warmblood horses with low TB proportions in their pedigrees [20, 21]. It is worth asking whether the level of contribution of TB genes in horses may impact their responses to the vocalizations of predators, i.e., a kind of a natural stimulus not familiar to the horses. A positive finding would be suggestive of a genetic effect in the horse’s response.

The effects of two predator species vocalizations were analyzed: gray wolf and Arabian leopard (*Panthera pardus nimr*). In Europe and northern Asia, packs of gray wolves were the main predators of large ungulates until recent ages, and horses of the studied breed coevolved with wolves but have had no recent exposure [22, 23]. After centuries of decline, wolf populations are now expanding in Europe [1]. In turn, leopards are distributed across southern Asia and sub-Saharan Africa and are critically endangered according to the Red List of Threatened Species of the International Union for Conservation of Nature [24, 25]. They avoid preying upon large animals, e.g., plains zebras [26]. Polish Halfbred horses have been evolutionarily isolated from leopards, and only the TB ancestors of Polish Halfbreds originating from Oriental horses could be familiar with leopard vocalizations. However, these Oriental ancestors were only imported in the 18th century [19]; hence, for the horses living in Europe a few centuries ago, the vocalizations of wolves were familiar, whereas those of leopards were alien. Currently, wolves are usually unfamiliar, and leopards are entirely strange to horses in Europe. When analyzing the effect of predation on modern horses, we aimed to compare the effects of predators that coevolved or were mostly isolated from the breed studied. The hypothesis was that horses were more frightened by a predator to which their ancestors were commonly exposed.

Auditory stimuli seem to be more effective than olfactory cues in eliciting responses from horses [27]. Since most predators do not vocalize while hunting, we used vocalizations typical for a species announcing their presence in a habitat: wolf howling and leopard growling [28]. The objective of the study was to determine the changes in cardiac activity, cortisol concentration, and behavior of horses in response to the predator vocalizations. In addition, we hypothesized that a higher proportion of TB ancestors in a horse’s pedigree resulted in higher emotional agitation and behavioral activity in response to predator vocalizations.

## Results

The analysis showed that the main effects: period of the test (period), proportion of TB ancestors in the pedigree (pedigree) and kind of predator (predator) significantly influenced most cardiac variables and cortisol levels (*p* < 0.05). The low-frequency component of the power spectrum (signal) assigned to the tone of the sympathetic nervous system (LF) was not significantly affected by the pedigree and predator factors. In addition, the predator did not significantly affect the root mean square of the successive differences in beat-to-beat intervals (RMSSD) or the high frequency component of the power spectrum assigned to the tone of the vagal nervous system (HF). The analysis focused on the period*pedigree*predator interaction which significantly influenced the variables apart from the ratio of LF to HF signal (LF/HF; *p* = 0.1400).

### Cardiac parameters that shift towards sympathetic nervous system activity

The influence of the period*pedigree*predator interaction on HR was statistically significant (*p* = 0.0494). The horse groups with various proportions of TB ancestry differed with regard to HR in the prestimulus period, with a lower HR observed in the ¾TB group compared to the other groups (*p* < 0.05; Table 1). During the stimulus period, all groups exhibited increased HR, whereas during the silent poststimulus period, HR was reduced (*p* < 0.05). The HRs of ½TB and ¼TB were higher in the scenario featuring leopard growling compared to that featuring wolf howling (*p* < 0.05). This difference remained in the poststimulus period.

**Table 1:** Cardiac activity parameters with shift towards sympathetic nervous system activity in horses at successive periods of the test of predator vocalization (means ± standard deviations)

Horse pedigree group	Prestimulus period	Stimulus period	Poststimulus period
HR during testing the wolf sound			
¾TB	39.0 ± 6.1 ax	50.7 ± 11.1 ay	39.3 ± 0.6 ax
½TB	49.0 ± 6.6 bx	57.0 ± 18.8 ay*	42.5 ± 7.1 ax*
¼TB	43.5 ± 4.1 cx*	52.0 ± 10.2 ay*	41.2 ± 4.0 ax*
HR during testing the leopard sound			
¾TB	44.0 ± 7.0 axy	51.2 ± 10.5 ay	39.4 ± 8.8 ax
½TB	57.2 ± 9.6 bx	75.3 ± 12.0 by	56.5 ± 7.5 bx
¼TB	55.0 ± 10.1 bx	67.3 ± 10.7 cy	54.7 ± 6.7 bx
LF during testing the wolf sound			
¾TB	3119 ± 886 ax	2909 ± 1403 ax*	2698 ± 763 ax*
½TB	3613 ± 2015 ax	6337 ± 1886 by*	4437 ± 1407 bz*
¼TB	4371 ± 1792 ax	2946 ± 981 ay	3770 ± 1148 cz*
LF during testing the leopard sound			
¾TB	3269 ± 2469 ax	6781 ± 2853 ay	1519 ± 638 az
½TB	3514 ± 2781 ax	4338 ± 1999 bx	2467 ± 1010 bx
¼TB	3396 ± 2394 ax	2680 ± 1634 cxy	2196 ± 1168 by

¾TB, ½TB, ¼TB – horse groups with 75%, 50% and 25% Thoroughbred ancestry within the parental and grandparental generations, respectively

*HR* heart rate, *LF *low frequency component of the power spectrum

Means marked with different letters significantly differ (according to Tukey’s HSD test) at *p* < 0.05: a, b, c - in columns (between different horse pedigree groups exposed to the same predator sound); x, y, z - in rows (between the same horse pedigree group at different test periods); *between the same horse pedigree group exposed to different predator sounds in analogical period of the test

The effect of the period*pedigree*predator interaction on LF was statistically significant (*p* = 0.0430). Significant differences (*p* < 0.05) in LF were observed between the groups during the prestimulus and stimulus periods, however they were not regular. The response to the predator sounds was not consistent: in some horse groups, the level of LF increased, and in some, it decreased (*p* < 0.05), whereas after the sounds stopped, LF usually decreased (*p* < 0.05). In the ¾TB group, LF was lower when the wolf howls were heard compared to the leopard growls, whereas in the ½TB group, the leopard growls elicited a weaker reaction (*p* < 0.05).

### Cardiac parameters that shift towards parasympathetic nervous system activity

The beat-to-beat intervals (RR) were significantly influenced by the period*pedigree*predator interaction (*p* = 0.0469). The RR was higher in the ¾TB group than in the other groups (*p* < 0.05; Table 2). The parameter usually decreased during the stimulus period and increased during the poststimulus period (*p* < 0.05). Within ½TB and ¼TB, RR was higher with wolf howling playback compared to with leopard growling playback (*p* < 0.05).

**Table 2:** Cardiac activity parameters with shift towards parasympathetic nervous system activity in horses at successive periods of the test of predator vocalization (means ± standard deviations)

Horse pedigree group	Prestimulus period	Stimulus period	Poststimulus period
RR during testing the wolf sound			
¾TB	1559 ± 223 ax	1232 ± 312 ay	1513 ± 17 ax
½TB	1292 ± 169 bx*	1136 ± 337 ax*	1444 ± 234 ay*
¼TB	1392 ± 129 bx*	1189 ± 270 ay	1469 ± 137 ax*
RR during testing the leopard sound			
¾TB	1431 ± 413 ax	1307 ± 431 ax	1575 ± 327 ax
½TB	1033 ± 150 bx	861.2 ± 206 by	1079 ± 140 bx
¼TB	1052 ± 179 bxy	909.3 ± 160 bx	1106 ± 148 by
RMSSD during testing the wolf sound			
¾TB	104.0 ± 25.3 ax	86.9 ± 17.5 ay*	101.8 ± 5.6 ax*
½TB	79.0 ± 27.9 ax*	91.1 ± 48.6 axy*	102.9 ± 37.0 ay*
¼TB	85.0 ± 19.5 ax*	70.5 ± 40.2 ax	81.1 ± 31.8 ax
RMSSD during testing the leopard sound			
¾TB	88.0 ± 26.4 ax	188.2 ± 136.3 ay	66.9 ± 18.5 abz
½TB	55.6 ± 23.8 bx	57.0 ± 51.2 bx	59.9 ± 21.6 ax
¼TB	59.6 ± 22.5 bx	54.8 ± 31.3 bx	71.0 ± 21.2 by
HF during testing the wolf sound			
¾TB	2700 ± 1238 ax	2048 ± 965 ax*	2582 ± 267 ax*
½TB	1596 ± 901 bx*	2395 ± 1271 ay*	2801 ± 1064 ay*
¼TB	1670 ± 603 bx*	1480 ± 866 bx	1709 ± 1621 bx
HF during testing the leopard sound			
¾TB	2412 ± 1832 ax	1227 ± 370 ay	993 ± 437 ay
½TB	792 ± 558 bx	1257 ± 242 ay	1008 ± 700 ax
¼TB	990 ± 697 bx	991 ± 330 ax	1763 ± 789 by

¾TB, ½TB, ¼TB – horse groups with 75%, 50% and 25% Thoroughbred ancestry within the parental and grandparental generations, respectively

*RR *beat-to-beat intervals, *RMSSD *root mean square of the successive differences in RR, *HF *high frequency component of the power spectrum

Means marked with different letters significantly differ (according to Tukey’s HSD test) at *p* < 0.05: a, b - in columns (between different horse pedigree groups exposed to the same predator sound); x, y, z - in rows (between the same horse pedigree group at different test periods); *between the same horse pedigree group exposed to different predator sounds in analogical period of the test

The RMSSD was significantly affected by the period*pedigree*predator interaction (*p* = 0.0067). When leopard growling was featured, the variable was higher in the ¾TB group during the prestimulus and stimulus periods and in the ¼TB group during the poststimulus period (*p* < 0.05). The RMSSD changed during successive periods (*p* < 0.05), but no tendency was observed within the horse groups. When the horses were exposed to leopard growling, the RMSSD was higher in the ¾TB group and lower in the ½TB group compared to the response to wolf howling (*p* < 0.05). At the poststimulus period following exposure to the leopard growls, the parameter was lower in the ¾TB and ½TB groups compared to with wolf howling (*p* < 0.05).

The influence of the period*pedigree*predator interaction on HF was statistically significant (*p* = 0.0041). Usually, the level of HF was higher in the ¾TB group and lower in the ¼TB group than in other groups (*p* < 0.05). The changes at successive periods of the test were not regular. HF was lower with exposure to leopard growling compared to exposure to wolf howling in most cases (*p* < 0.05).

### Cortisol levels

**Fig. 1:**
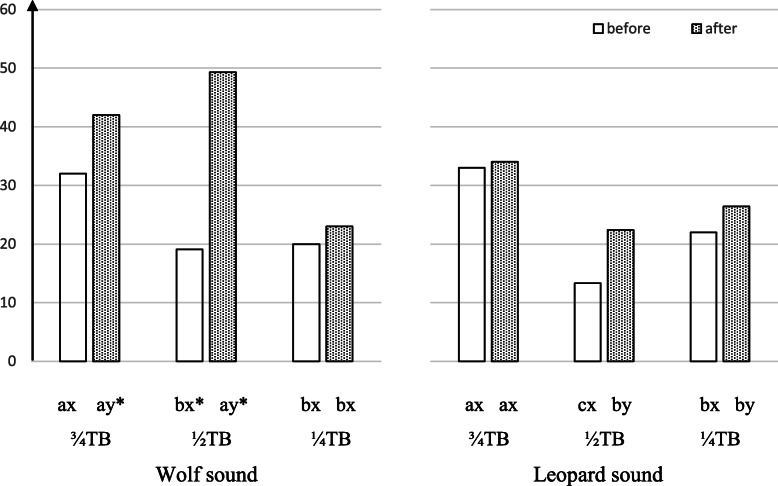
Cortisol concentration in horse saliva (means; µg/L) before and after exposure to predator vocalizations. Means marked with different letters significantly differ (according to Tukey’s HSD test) at *p* < 0.05: a, b, c -between different horse pedigree groups exposed to the same predator sound; x, y - between the same horse pedigree group at different test periods; * - between the same horse pedigree group exposed to different predator vocalizations ¾TB, ½TB, ¼TB – horse groups with 75%, 50% and 25% Thoroughbred ancestry within the parental and grandparental generations, respectively

The cortisol levels were significantly affected by the period*pedigree*predator interaction (*p* = 0.0394). Some differences were noted when the subjects were at rest; for example, in the ¾TB group, the cortisol levels were lower than in other cases (Fig. 1). In most cases, the cortisol levels were higher after the test than at rest and when the wolf was heard compared to with exposure to leopard vocalizations.

### Scores for significant differences in cardiac activity and cortisol variables

The scores assigned to assess the statistically significant changes in the cardiac activity and cortisol variables indicated that TB ancestry influenced the physiological responses of horses to predator vocalizations (Table 3). The ¾TB group was assigned a score of seven points; the ½TB group, four points; and the ¼TB group, two points. However, considering all possible changes across all variables, the differences among groups were relatively low. The response to wolf howling induced five significant changes, whereas leopard growling elicited eight significant changes.

**Table 3:** Scores for statistically significant differences in cardiac activity and cortisol variables within groups. A horse group could achieve a sum of scores ranging from − 11 to 11 for the response to vocalization of one predator

		Sympathetic activity				Parasympathetic activity						Cortisol	Total
Variable:		HR		LF		RR		RMSSD		HF			
Period:		2	3	2	3	2	3	2	3	2	3	3	
Horse pedigree group			Wolf sound										
¾TB		1				1		1				1	4
½TB		1		1	1				-1	-1	-1	1	1
¼TB		1		-1	-1	1							0
Horse pedigree group			Leopard sound										
¾TB	1			1	-1			-1	1	1	1		3
½TB	1		1			1				-1		1	3
¼TB	1				1	1			-1		-1	1	2

¾TB, ½TB, ¼TB – horse groups with 75%, 50% and 25% Thoroughbred ancestry within the parental and grandparental generations, respectively

*HR *heart rate, *LF *low frequency component of the power spectrum, ratio of LF and HF signal, where HF is a high frequency component of the power spectrum, *RR *beat-to-beat intervals, *RMSSD *root mean square of the successive differences in RR, *HF* high frequency component of the power spectrum

Period 2 – stimulus period; Period 3 – poststimulus period

### Behavior

The horses did not flee or reveal highly increased vigilance in response to the predator vocalizations. As presented in Tables 4 and 5, the horses rarely showed locomotor behavior, and no horses demonstrated increased vigilance during the prestimulus period. Immediately after the start of the stimulus period, the behavior changed as follows: 31–42% of horses walked, 10–26% trotted, and 0–10% galloped. Increased vigilance, which was expressed by the orientation of head towards the source of the sound, was shown by 53–89% of the horses, and elevated necks were shown by 36–79%, elevated tails by 21%, and alarm snorts by 0–16%. In the poststimulus period, directly after the stimulus was stopped, both locomotor and vigilance behaviors were again observed, although they were somewhat rarer than at the beginning of the stimulus period. Approximately one-third of the differences in the percentage of horses showing a behavior between the successive periods of the test and one-quarter of the differences between the various TB groups were significant (*p* < 0.05). Considering all the horses in the stimulus and poststimulus periods together, locomotor behaviors were recorded 1.8 times more frequently and increased vigilance was 1.7 times more frequent when the horses were exposed to leopard growls than to wolf howls, and the percentage differences were significant in half of the cases. Significant differences (*p* < 0.05) in the percentages of behaviors in response to the two predators’ sounds also appeared in half of the cases.

**Table 4:** The percentage of horses in a group showing locomotor behaviors during the first 30 s of a test period

Horse pedigree group	Prestimulus period	Stimulus period		Poststimulus period
Wolf sound				
Walk				
¾TB	17ax	33ay	17ax	
½TB	28bx	14by	0bz	
¼TB	0cx	50cy	17az	
Trot				
¾TB	0ax	17ay	0ax	
½TB	0ax	14ay	0ax	
¼TB	0ax	0bx	0ax	
Gallop				
¾TB	0ax	0ax	0ax	
½TB	0ax	0ax	0ax	
¼TB	0ax	0ax	0ax	
Leopard sound				
Walk				
¾TB	0ax*	33ay	33ay*	
½TB	0ax*	43ay*	43ay*	
¼TB	0ax	17ay*	17ay*	
Trot				
¾TB	17ax*	17ax	17ax*	
½TB	0bx	28ay*	43by*	
¼TB	0bx	33ay*	0cx	
Gallop				
¾TB	0ax	17ay*		17ay*
½TB	0ax	33ay*		0bx
¼TB	0ax	17ay*		0bx

¾TB, ½TB, ¼TB – horse groups with 75, 50 and 25% Thoroughbred ancestry within the parental and grandparental generations, respectively

Percentages marked with different letters significantly differ (according to Parker’s test) at *p* < 0.05: a, b, c - in columns (between different horse pedigree groups exposed to the same predator sound); x, y, z - in rows (between the same horse pedigree group at different test periods); *between the same horse pedigree group exposed to different predator sounds in analogical period of the test

**Table 5:** The percentage of horses in a group showing increased vigilance during the first 30 s of a test period

Horse pedigree group	Prestimulus period	Stimulus period	Poststimulus period
Wolf sound			
Head oriented towards the source of the sound			
¾TB	-	33ax	17ay
½TB	-	71bx	28aby
¼TB	-	50abx	33bx
Elevated neck			
¾TB	0ax	50ay	17ay
½TB	0ax	43ay	14az
¼TB	0ax	67ay	33cz
Elevated tail			
¾TB	0ax	33ay	0ax
½TB	0ax	28ay	0ax
¼TB	0ax	0bx	0ax
Vocalization – alarm snort			
¾TB	0ax	0ax	0ax
½TB	0ax	0ax	0ax
¼TB	0ax	0ax	0ax
Leopard sound			
Head oriented towards the source of the sound			
¾TB	-	83ax*	50ay*
½TB	-	86ax	100bx*
¼TB	-	100ax*	67by*
Elevated neck			
¾TB	0ax	50ay	33ay*
½TB	0ax	100by*	100by*
¼TB	0ax	83by	33az
Elevated tail			
¾TB	0ax	0ax*	0ax
½TB	0ax	28by*	14bxy
¼TB	0ax	33by*	17bz*
Vocalization – alarm snort			
¾TB	0ax	0ax	0ax
½TB	0ax	14by*	0ax
¼TB	0ax	33cy*	0ax

¾TB, ½TB, ¼TB – horse groups with 75%, 50% and 25% Thoroughbred ancestry within the parental and grandparental generations, respectively

(-) lack of stimulus at the prestimulus period

Percentages marked with different letters significantly differ (according to Parker’s test) at *p* < 0.05: a, b, c - in columns (between different horse pedigree groups exposed to the same predator sound); x, y, z - in rows (between the same horse pedigree group at different test periods); *between the same horse pedigree group exposed to different predator sounds in analogical period of the test

## Discussion

The present study was the first attempt to test whether sounds of predators are frightening to horses bred by humans and to assess the responses the sounds elicited. The results indicated that the horses reacted to the studied predator vocalizations, but the responses were weak and heterogeneous, i.e. the changes in the variables not always underwent in one direction. The cardiac activity variables studied are commonly regarded as indicators of a horse’s emotional status [29]. For instance, an intensive fear response is characterized by a strong increase in HR [11]. This increase in HR is a typical reaction observed in horses in response to novelty [29, 30]. Although HR increased in all cases in response to predator vocalizations in the present study, the changes in this variable were lower than those in the above-cited papers. These HR levels suggested that the horses were more interested in the sound rather than frightened. The LF and LF/HF reflecting a shift towards sympathetic nervous system activity did not change in a certain direction (LF/HF was not a significant effect), similar to RMSSD and HF. Moreover, the high standard deviations for RMSSD, LF and HF indicated that there were great individual differences between horses. The RR generally decreased, reflecting a decrease in vagal nervous system activity during the experiment. In studies on the treatment of physiotherapy and other relaxation methods used in horses, this parameter was found to increase [31, 32]. The saliva cortisol concentration significantly increased in most cases in the present study, which indicated that some level of stress in horses is related to their emotional excitability [7].

Changes in behavior illustrate the external response of an animal to a fear- or stress-eliciting factor [4]. The predator vocalizations seemed to be too weak of a stimulus to induce intense fear and consequently a flight response, instead eliciting only a short-term increase in locomotor behavior and vigilance. Ceasing the stimulus resulted in a less intense but similar short-term response to that elicited by the playback, which may be interpreted as the sudden silence disquieting the horses.

Regardless of the external observations, the internal responses to the predator sounds, which were expressed by the increased HR and cortisol levels, as well as a decrease in RR, evidently implied some level of stress in the horses. Similar findings with regard to HR and behavior in horses’ responses to the presence of predator odor, expressed together with a sudden auditory stimulus, were described by Christensen and Rundgren [10]. The odor *per se* did not frighten horses but did cause an increase in vigilance. It seems that in the past, when equids shared habitats with numerous predators, equids could recognize predators very well, and their response to predation was sufficient, given that at least *Equus caballus* survived. Since predator cues do not elicit strong responses currently, it means that domestic horses do not recognize threats well and that their abilities to raise adequate responses are attenuated.

As we hypothesized, due to the sensitivity of TB, a higher prevalence of TB ancestry should elicit a stronger response to predator vocalizations. The scores for significant changes in the cardiac and cortisol variables confirmed that the responses were stronger to some extent in the horses with the highest proportion of TB ancestry and weaker in the groups with lower proportions. Thus, a tendency towards a stronger internal reaction to predators exists in horses with a higher proportion of TB genes, which suggests that the response intensity is partly genetically coded. This result is consistent with that of Von Borstel et al. [21], who documented that warmbloods with a high proportion of TB ancestry were more reactive to novel stimuli, including auditory cues, than those with a lower proportion of TB ancestry. Budzyńska et al. [20] also found an increased fear reaction in stallions with a higher proportion of TB ancestry. Stallions of over 75% TB ancestors revealed higher HR before the fearfulness test and needed more time to pass novel objects.

The number of significant changes in the variables studied, as well as the frequency of locomotor behaviors and vigilance intensity, indicated a tendency against our hypothesis that the wolf vocalization would be more frightening than that of the leopard. Instead, the leopard growl elicited a stronger response. As has been mentioned, the horses had not had any contact with wolves or leopards previously, although their ancestors presumably were familiar with wolf howling. The observed significant changes in the variables showed a weak tendency of a stronger response by the horses with the highest proportions of TB ancestry than the other horse groups to leopard growls compared to the wolf howls. The differing responses to the two predator species might suggest that animals were more frightened of an unknown threatening predator sound than of a sound known by their ancestors. Despite the fact that both vocalizations had similar equivalent continuous sound levels (LAeq), the lower amplitude of growls compared to howls may have also partly caused the stronger reaction to the leopard vocalization. According to Morton’s hypothesis on motivational-structural rules [33] developed by August and Anderson [34], aggressive sounds like the growl are of low-frequency and wide bandwidth, whereas high-frequency and tonal sounds may convey motivation information in fearful or friendly contexts. Morton [33] qualified barking as neither aggressive nor fearful or appeasing cue. The low amplitude of the leopard growl could mean that the predator’s tranquility was dismayed and the predator is in close proximity, hence an attack may be imminent. If horses could discriminate the type of predator vocalization, they would be more scared by a leopard present in the vicinity than by wolf howling heard from a distance. Differing behavioral responses to vocalizations of different predators have been reported in wild animals, e.g., mule deer or elephants being able to discriminate the type of danger [28, 35]. However, more testing is needed to document such suggestions regarding domestic horses.

In summary, heterogeneous physiological and behavioral changes in response to predator vocalizations indicated low sensitivity to these sounds in present-day horses. At this level of response intensity, a tendency towards a stronger internal reaction to predators in horses with a higher proportion of TB genes was weakly expressed. The external response was less distinct than the internal physiological changes, which may imply that the horses handled and trained by humans are able to manage their emotions. Domestication aimed to tame horses and to prompt them to develop the ability to detect human signals. Horses became more dependent on humans and are now presumably less able to react appropriately to problems by themselves [36]. They are trained to be habituated to novel objects, which reduces their responses [12]. To attenuate specific unwanted behaviors, they are exposed to behavioral modification techniques [13]. The demand for horses qualified to be used in various branches of the horse industry directed the aims of horse breeding towards specific proprieties while simultaneously reducing the intensity of the horse’s responses to fear-inducing stimuli.

## Conclusions

Despite the fact that predator vocalizations are usually unfamiliar to present-day domestic horses, the horses responded with signs of anxiety when exposed to such auditory stimuli. The response was weak compared to, for instance, the novelty test described in the referenced literature. The tendency towards a stronger internal reaction to predators in horses with a higher proportion of TB genes suggested that the response intensity was partly innate. The response was elicited both by the wolf, which horses coevolved with but had no recent exposure to, and by the Arabian leopard from which the horses have been mostly isolated. The more pronounced response to the leopard may indicate that horses were more frightened of an unknown threatening predator sound than by one known to their ancestors. The differing response can be also due to differences in the characteristic of the predators’ vocalizations. Our findings suggested that the present-day horses’ abilities to coexist with predators are weak presumably due to domestication, which tamed the horses and was followed by long-term selection for utility traits. Hence, a prospective expansion of the gray wolf population might be dangerous for horses in the future, and humans should protect horses against predation, especially when introducing them into seminatural locations.

## Methods

### Horses

Nineteen leisure horses (seven mares and 12 geldings) were included in the study. They were from six to 10 years old (mean 89.1 ± 12.3 months). They were all representatives of Polish Halfbred horses and originated from matings of German horses with Wielkopolski warmblood breeds; they had various percentages of TB ancestry in their pedigrees. The horses were confirmed to be clinically sound. A person test [37] performed by a behaviorist did not detect any behavioral disturbances or excessive excitability. The mares did not show external signs of estrus. The horses belonged to the University of Life Sciences in Lublin and were maintained in the university’s experimental facility, which is located close to urban surroundings where no cases of predation have been noticed. The horses were kept in stable box stalls, released into paddocks for five hours each day and ridden for two hours per day.

### Experimental procedure

The horses were divided into three groups according to the percentage of TB ancestry in the pedigree within the parental and grandparental generations: six horses of 75% (¾TB), seven horses of 50% (½TB) and six horses of 25% (¼TB) were assessed. Pure TBs were not included in the study since they are typically different from leisure horses. Each TB group was randomly divided into halves: A subgroup and B subgroup. For the four weeks prior to the beginning of the experiment, the horses were turned out into paddocks in these respective groups. The test was conducted in a 30 × 40 m experimental paddock that was familiar to the horses and located 500 m from the stable This distance prevented the predator sounds featured in the test from being heard in the stable. The earthen paddock was situated away from roads, noises, or other stressors and did not contain any food. Each individual group of horses was first turned out into another paddock located behind the stable to reduce agitation after the time spent in the stable and after one hour was released into the experimental paddock brought there in hand.

The experiment was conducted in April (12 °C, cloudy without precipitation, and wind speed below 0.5 m/s). On the first day of the experiment, the three A subgroups were assigned to the wolf vocalization trials, which were conducted successively at 9:00, 9:30 and 10:00. The other three halves (B subgroups) were tested at 10:30, 11:00 and 11:30 using the leopard playback. The next day, the subgroups were released into the experimental paddock in the same order but without featuring the playback. On the third day, the procedure without the stimulus was repeated; however, subgroups B were turned out before subgroups A. On the fourth day, the subgroups were tested again in the same order as a day before, but subgroups B could hear the wolf and subgroups A heard the leopard. There was a two-day pause in the test (2nd and 3rd days of the experiment) when the horse groups were released into the experimental paddock and the stimulus was not used, which should have prevented the horses from becoming habituated to the predator vocalization.

The sounds consisted of a gray wolf howling and an Arabian leopard growling (resembling a sawing roar), which were obtained from commercial CDs. The source of the sound was placed outside the paddock in the fence corner closest to the stable corner. The playback was emitted by a two-way loudspeaker characterized by a rated power of 25 W and a speaker frequency response ranging from 20 to 20,000 Hz (Sony CMT-SBT100). The levels and frequency ranges of the sounds were measured using a sonometer DT-8852 (CEM, Poland). The measurements were made at a distance of 1 m from the source of the loudspeaker over the entire duration of sound played in the experiment. The A-weighted LAeq was measured in decibels. The sounds produced by the wolf and leopard had similar LAeq values (58.6 and 55.9 dB, respectively). LAFmin and LAFmax were the minimum and maximum values of LAeq in the fast response, respectively. The LAFmin values were 43.6 and 37.7 dB, and the LAFmax values were 65.7 and 82.5 dB for the wolf and leopard, respectively. The maximum sound pressure level was 1000 Hz in both cases. The wolf howls were dominated by low frequency sounds (250–2000 Hz), whereas the leopard growls had a wider frequency range (250–5000 Hz).

The testing phase comprised three periods that lasted a total of 25 minutes: a 10-min prestimulus period without any experimental stimuli, a 5-min stimulus period when a predator sound was heard, and a 10-min poststimulus period without any experimental stimuli. After completion of the test, the horses were led to the stable, where saliva samples were collected. They were used for leisure riding in the afternoon, as before the experiment. The clinical examination and person test were performed again and did not show any changes in physical state or behavior of the horses one day after the experiment.

### Measurement and elaboration of data

The response to the test was mainly considered in terms of the emotional state of the horse, and observations of the behavioral responses provided an additional aspect. Three kinds of variables were collected: (1) heart activity variables were monitored continuously over the test duration; (2) the cortisol concentrations were determined in the saliva sampled two times each day: at rest at 07:00 and 15 min after the poststimulus period; and (3) the percentage of horses that showed locomotor behavior and increased vigilance during the first 30 s of each period. The cardiac activity was registered with a telemetric Polar ELECTRO OY-RS800CX device [38]. The horses were habituated to the device for six days prior to the start of the experiment. The device was synchronized with a stopwatch to enable monitoring heart activity changes during the successive periods of the test. The data were downloaded to a computer with a peripheral IrDA USB 2.0 adapter and analyzed with PolarProTrainer5 Kubios HRV software, version 2.0 [39, 40], which made it possible to correct the electrocardiogram record. Heart function was measured with HR (beats per minute) and heart rate variability (HRV) parameters: RR (ms), RMSSD (ms), LF (ms^2^), HF (ms^2^), and LF/HF (%). HR, LF, and LF/HF increase as a result of sympathetic nervous system activity, whereas RR, RMSSD, and HF reflect parasympathetic (vagal) modulation activity.

Saliva samples were collected with a small piece of sponge inserted into the horse’s mouth. After soaking up saliva, the sponge was placed in a plastic tube and stored at -20 °C. The laboratory analysis was conducted using the enzyme-immunoassay method with the ELISA SLV-4635 kit (Diagnostic System Laboratories Inc., Webster, TX, USA) as described by Kędzierski et al. [41]. The absorbance was determined with a multiscan reader (Labsystem, Helsinki, Finland) with the use of Genesis v.3.00 software and amounted to 450 ± 10 nm. The intra and interassay coefficients of variation for salivary cortisol determined in the laboratory amounted to 8% and 11%, respectively.

The turnouts were continuously recorded on video for subsequent analysis of the horses’ behavior. We focused on behaviors that might illustrate fear in a paddock without food, i.e., locomotor behaviors (walk/trot/gallop) and/or increased vigilance (head oriented towards the source of the sound/elevated neck/elevated tail/vocalization - alarm snort; Table 6) [4, 42]. More than three steps without interruption was recorded as a gait. The number of horses that showed a behavior at least once was recorded, irrespective of the duration and intensity of the behavior. Finally, only behaviors observed during the first 30 s of each period were considered, since the horses generally showed them immediately after beginning of the stimulus and poststimulus periods.

**Table 6:** Ethogram of behaviors indicating increased vigilance [4, 42]

Behavior	Description
Head oriented towards the source of the sound	Horse standing with weight resting on four limbs; head, ears and eyes oriented towards the stimulus
Elevated neck	Neck raised over 45 degrees; head scanning the surroundings
Elevated tail	Fleshy part of tail outstretched horizontally or elevated above horizontal
Alarm snort	Short powerful exhalations from nostrils

### Statistical analysis

The experiment included three factors with repeated measures within one of the factors. Before a method of statistical analysis was chosen, the data were checked using the Shapiro-Wilk test. No inconsistency with the normal distribution was demonstrated. Levene’s test showed the homogeneity of variances (*p* > 0.05). The hypothesis of the study related to the period was tested by a multivariate analysis of variance (MANOVA) using SAS version 9.1.3 (SAS Institute, Cary, NC, USA). The following main factors were taken into consideration: period which consisted of the repeated measures during the test (prestimulus, stimulus, poststimulus); pedigree, i.e. the proportion of TB ancestry in a horse’s pedigree (¾TB, ½TB, ¼TB); and predator (wolf howls, leopard growls), as well as interactions between these factors. The results from A and B subgroups were considered in total within the TB groups. After rejection of the null hypothesis, a post-hoc comparison of the means was performed using Tukey’s HSD test taking into account different numbers of horses in groups. Differences between means at *p* < 0.05 were considered statistically significant. In addition, standard deviation (sd) was calculated.

To make the physiological results of the analysis easily interpretable, i.e., to determine whether the changes were directed towards the sympathetic or parasympathetic nervous system, we used a modified evaluation score scale according to Kędzierski et al. [41]. Specifically, we assigned one point for a statistically significant increase in the parameters assigned to sympathetic system activity (HR, LF) in the stimulus and poststimulus periods compared to in the prestimulus period and cortisol after the poststimulus period compared to at rest. Similarly, one point was given when parameters associated with vagal nervous system activity (RR, RMSSD, HF) significantly decreased during the study. When a decrease in LF was observed or an increase in RMSSD and HF occurred, a negative point was given.

The percentage of horses in a group that showed a specific behavior was calculated. Significant differences between the percentages of horses in the groups were determined at *p* < 0.05 with the use of Parker’s test [43].

## Data Availability

The datasets used and analyzed during the current study are available from the corresponding author on reasonable request.

## Abbreviations

TB: Thoroughbred horse
HR: heart rate
period: period of the test
pedigree: proportion of TB ancestors in the pedigree
predator: kind of predator
LF: low frequency component of the power spectrum assigned to the tone of the sympathetic system
RMSSD: root mean square of the successive differences in RR
HF: high frequency component of the power spectrum assigned to the tone of the vagal system
LF/HF: ratio of LF to HF
RR: beat-to-beat intervals
LAeq: A-weighted equivalent continuous sound level
LAFmin and LAFmax: minimum and maximum values of LAeq in fast response
HRV: heart rate variability
sd: standard deviation
